# Metabolic Responses to Polymyxin Treatment in *Acinetobacter baumannii* ATCC 19606: Integrating Transcriptomics and Metabolomics with Genome-Scale Metabolic Modeling

**DOI:** 10.1128/mSystems.00157-18

**Published:** 2019-02-05

**Authors:** Yan Zhu, Jinxin Zhao, Mohd Hafidz Mahamad Maifiah, Tony Velkov, Falk Schreiber, Jian Li

**Affiliations:** a Infection & Immunity Program, Monash Biomedicine Discovery Institute and Department of Microbiology, Monash University, Melbourne, Australia; b Drug Delivery, Disposition and Dynamics, Monash Institute of Pharmaceutical Sciences, Monash University, Melbourne, Australia; c Department of Pharmacology and Therapeutics, University of Melbourne, Melbourne, Australia; d Department of Computer and Information Science, University of Konstanz, Konstanz, Germany; University of Illinois at Chicago

**Keywords:** *Acinetobacter baumannii*, genome-scale metabolic modeling, metabolomics, transcriptomics, polymyxins

## Abstract

Multidrug-resistant (MDR) Acinetobacter baumannii has emerged as a very problematic pathogen over the past decades, with a high incidence in nosocomial infections. Discovered in the late 1940s but abandoned in the 1970s, polymyxins (i.e., polymyxin B and colistin) have been revived as the last-line therapy against Gram-negative “superbugs,” including MDR A. baumannii. Worryingly, resistance to polymyxins in A. baumannii has been increasingly reported, urging the development of novel antimicrobial therapies to rescue this last-line class of antibiotics. In the present study, we integrated genome-scale metabolic modeling with multiomics data to elucidate the mechanisms of cellular responses to colistin treatment in A. baumannii. A genome-scale metabolic model, *i*ATCC19606, was constructed for strain ATCC 19606 based on the literature and genome annotation, containing 897 genes, 1,270 reactions, and 1,180 metabolites. After extensive curation, prediction of growth on 190 carbon sources using *i*ATCC19606 achieved an overall accuracy of 84.3% compared to Biolog experimental results. Prediction of gene essentiality reached a high accuracy of 86.1% and 82.7% compared to two transposon mutant libraries of AB5075 and ATCC 17978, respectively. Further integrative modeling with our correlative transcriptomics and metabolomics data deciphered the complex regulation on metabolic responses to colistin treatment, including (i) upregulated fluxes through gluconeogenesis, the pentose phosphate pathway, and amino acid and nucleotide biosynthesis; (ii) downregulated TCA cycle and peptidoglycan and lipopolysaccharide biogenesis; and (iii) altered fluxes over respiratory chain. Our results elucidated the interplay of multiple metabolic pathways under colistin treatment in A. baumannii and provide key mechanistic insights into optimizing polymyxin combination therapy.

**IMPORTANCE** Combating antimicrobial resistance has been highlighted as a critical global health priority. Due to the drying drug discovery pipeline, polymyxins have been employed as the last-line therapy against Gram-negative “superbugs”; however, the detailed mechanisms of antibacterial killing remain largely unclear, hampering the improvement of polymyxin therapy. Our integrative modeling using the constructed genome-scale metabolic model *i*ATCC19606 and the correlative multiomics data provide the fundamental understanding of the complex metabolic responses to polymyxin treatment in A. baumannii at the systems level. The model *i*ATCC19606 may have a significant potential in antimicrobial systems pharmacology research in A. baumannii.

## Abstract (precis)

Combating antimicrobial resistance has been highlighted as a critical global health priority. Due to the drying drug discovery pipeline, polymyxins have been employed as the last-line therapy against Gram-negative “superbugs”; however, the detailed mechanisms of antibacterial killing remain largely unclear, hampering the improvement of polymyxin therapy. Our integrative modeling using the constructed genome-scale metabolic model *i*ATCC19606 and the correlative multiomics data provide the fundamental understanding of the complex metabolic responses to polymyxin treatment in A. baumannii at the systems level. The model *i*ATCC19606 may have a significant potential in antimicrobial systems pharmacology research in A. baumannii.

## INTRODUCTION

Acinetobacter baumannii is a very problematic opportunistic Gram-negative pathogen with a high mortality in critically ill patients (1, 2). It causes a range of nosocomial infections, including pneumonia, bacteremia, urinary tract infections, and meningitis (3, 4). A. baumannii can rapidly develop resistance to multiple antibiotics via acquiring heterogeneous genetic materials (5) or spontaneous mutagenesis (6, 7). Recently, the World Health Organization prioritized carbapenem-resistant A. baumannii as one of the three “Critical” bacterial pathogens that urgently require development of novel antibiotics (http://www.who.int/medicines/publications/global-priority-list-antibiotic-resistant-bacteria/en/).

Discovered in the 1940s, polymyxins waned in the 1970s due to their potential nephrotoxicity and neurotoxicity (8). Until the last decade, they have been revived as the last-line therapy against Gram-negative “superbugs,” including MDR A. baumannii (8). Polymyxins are amphipathic, nonribosomal synthesized lipodecapolypeptides containing five positively charged l-α,γ-diaminobutyric acid residues (8). The purported primary mechanism of polymyxin activity involves initial polar and hydrophobic interactions with lipid A of lipopolysaccharide (LPS) in Gram-negative bacterial outer membrane (OM), followed by the displacement of calcium (Ca^2+^) and magnesium (Mg^2+^), and OM disorganization (8). Alternative secondary antibacterial mechanisms were proposed, including through hydroxyl radical production and inhibition of the inner membrane (IM) respiratory enzymes (e.g., type II NADH-quinone oxidoreductase) (9, 10). Resistance to polymyxins in A. baumannii is mainly due to lipid A modifications (with phosphoethanolamine or galactosamine by *pmrC* and *naxD*, respectively) or LPS loss (mediated by mutations in *lpxACD*) (11–13). Several studies reported that metabolic changes in pentose phosphate pathway (PPP) and glycerophospholipid and peptidoglycan biosynthesis are associated with polymyxin resistance in A. baumannii (14, 15). Nevertheless, there is limited information on the complex metabolic network that controls responses to polymyxin killing in A. baumannii.

A genome-scale metabolic model (GSMM) assembles all biochemical reactions possibly occurring in an organism to predict cellular metabolic functions (16), delineate the mechanisms of antimicrobial killing (17, 18), and facilitate drug discovery (19). GSMMs also provide systems platforms for integrative analysis of multiomics data (17, 20–22). Currently, there are only three existing GSMMs for A. baumannii, AbyMBEL891 and iCN718 for strain AYE (23, 24), and iLP844 for ATCC 19606 (25). However, AbyMBEL891 lacks experimental validation for nutrient utilization prediction and employed a non-A. baumannii mutant library to assess the predicted gene essentiality (23). Models iCN718 and iLP844 used 78 and 64 carbon sources, respectively, to validate nutrient utilization prediction (24, 25). Model iLP844 integrated transcriptomics data (untreated control and colistin treatment at 2 mg/liter for 15 and 60 min) to simulate metabolic changes under treatment using the Metabolic Adjustment by Differential Expression (MADE) algorithm (25, 26). However, MADE relies on simple discretization of transcriptomic data and potentially imprecisely represents continuous gene expression profiles, thereby leading to inaccurate predictions (27). Moreover, apart from transcriptional regulation, many intermediate steps (e.g., metabolic regulation) may jointly affect the overall metabolic activity. Therefore, further integrative analysis with metabolomics data will be crucial for delineating the regulation.

Here we report the development and validation of a GSMM for A. baumannii ATCC 19606 using the literature, genome annotation, and experimental data from our laboratory. Integrative analysis with transcriptomics and metabolomics data revealed that the PPP, glyoxylate shunt, arginine biosynthesis, and LPS and peptidoglycan biosynthesis played key roles in metabolic responses to colistin treatment. The simulation results provide novel mechanistic insights into developing synergistic polymyxin combinations to combat MDR A. baumannii.

(Part of this work was presented at the 13th Annual Conference of the Metabolomics Society, 25 to 29 June 2017, Brisbane, Australia.)

## RESULTS

### Construction of the genome-scale metabolic model *i*ATCC19606.

**FIG 1:**
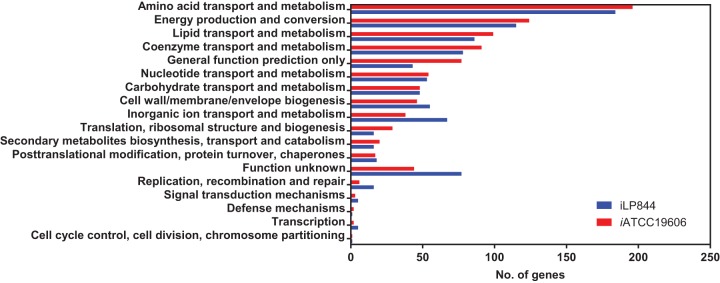
The COG functional classification of the involved genes in *i*ATCC19606 and iLP844.

To expedite the GSMM construction, a draft model was developed using AbyMBEL891 as a template (23). First, 3,078 orthologs were identified between strains AYE and ATCC 19606 using reciprocal BLASTp with identity of >70%, E value of <1E−5, and coverage of >70% (28). AbyMBEL891 contained 650 genes in A. baumannii AYE, and 630 of them were replaced with their corresponding orthologs in ATCC 19606 (23). Five AYE-specific enzymatic reactions were deleted due to the lack of their counterparts in ATCC 19606. Second, the ATCC 19606 genome was annotated using KEGG (Kyoto Encyclopedia of Genes and Genomes) BlastKOALA (29), and 1,558 were assigned with KEGG Orthology. The draft model was supplemented with 231 metabolites, 218 reactions, and 164 genes from KEGG and MetaCyc (30). Extensive manual curation was conducted to fill pathway gaps. Transport and exchange reactions were added, enabling nutrient uptake and by-product secretion. Finally, the resulting model was designated *i*ATCC19606 and involved 1,180 metabolites, 1,270 reactions, and 897 genes, representing 23.4% of the entire ATCC 19606 genome (Table 1; see also Data Set S1A and Data Set S2 in the supplemental material). Among the 23 Clusters of Orthologous Groups (COGs), 18 were covered by *i*ATCC19606 (Fig. 1). The top three largest COG classes include amino acid metabolism, energy production and conversion, and lipid metabolism. *i*ATCC19606 and iLP844 (25) shared 639 genes in common (Data Set S1B). Specifically, *i*ATCC19606 exclusively incorporated 258 genes mainly from energy production, metabolism of amino acids, lipids, and coenzymes (Fig. 1).

**TABLE 1:** Genome contents and model components

Content	Data for strain and model		
	AYE; AbyMBEL891	ATCC 19606	
		iLP844	*i*ATCC19606
Genome size (Mb)	4.04	3.97^*a*^	3.95
Assembly status	Complete	Contigs	Contigs
GenBank accession number(s)	CU459137–CU459141	ACQB00000000	JMRY00000000
GC content (%)	39.34	39.10	39.12
No. of genes	3,900	3,803	3,804
No. of CDS	3,703 (77^*b*^)	3,637 (102^*b*^)	3,669 (0^*b*^)
No. of contigs	-^*c*^	100	18
No. of reactions	891	1,628	1,270
No. of metabolites	778	1,509	1,180
No. of involved genes	650	844	897

a Different sizes of ATCC 19606 draft assemblies.

b Number of pseudogenes.

c -, complete genome with 1 chromosome and 4 plasmids.

10.1128/mSystems.00157-18.1 DATA SET S1 (A) Metabolites and reactions in *i*ATCC19606. (B) Gene contents in models *i*ATCC19606 and iLP844. (C) Prediction of growth on various carbon sources. (D) Predicted essential genes on five different media. (E) Comparison of predicted essential genes with AB5075 mutant library. (F) Comparison of predicted essential genes with ATCC 17978 mutant library. (G) Predicted essential reactions on five different media. (H) Predicted essential metabolites on five different media. (I) Predicted metabolic fluxes under control and 2-mg/liter colistin treatment. (J) Comparison of calculated flux sums with differential regulated metabolites. (K) Colistin MICs of mutants from AB5075 transposon insertion library. (L) Comparisons of reactions in iLP844 and *i*ATCC19606. (M) Nutrient uptake constraints for growth media. Download Data Set S1, XLS file, 2.3 MB. Copyright © 2019 Zhu et al. 2019 Zhu et al. This content is distributed under the terms of the Creative Commons Attribution 4.0 International license.

10.1128/mSystems.00157-18.2 DATA SET S2 *i*ATCC19606 in SBML format. Download Data Set S2, TXT file, 1.7 MB. Copyright © 2019 Zhu et al. 2019 Zhu et al. This content is distributed under the terms of the Creative Commons Attribution 4.0 International license.

### Prediction of bacterial growth on various nutrients.

**FIG 2:**
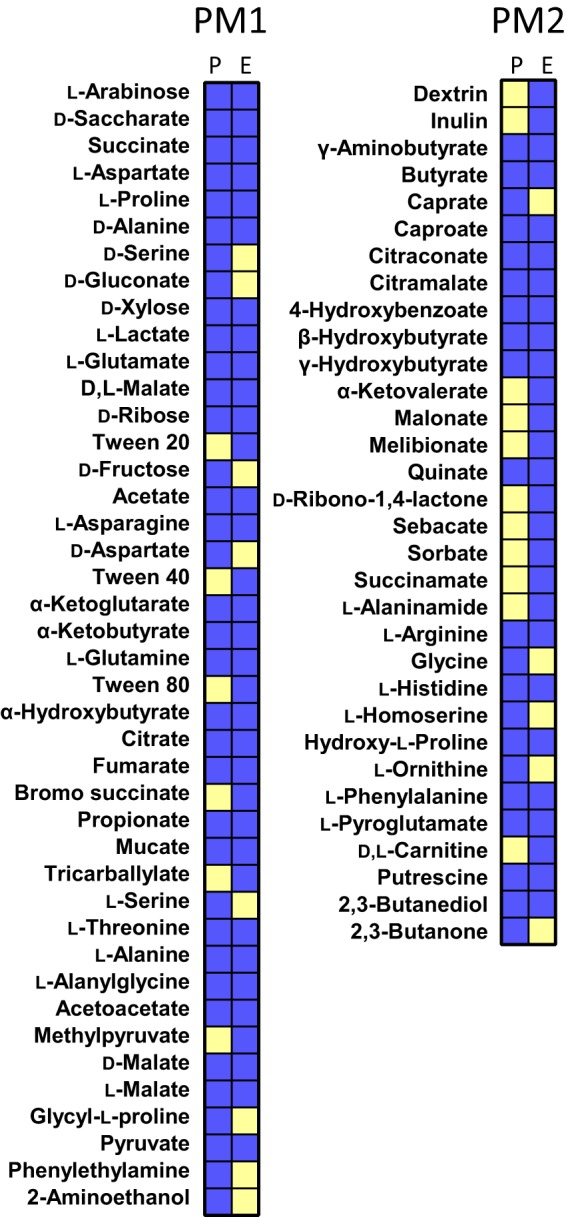
Comparison of the Biolog result (left columns, denoted by E) and model prediction (right columns, denoted by P). Blue indicates valid growth, and yellow indicates no growth. Only those carbon sources with a valid predicted and/or experimental growth were displayed.

Using flux balance analysis (FBA), *i*ATCC19606 predicted bacterial exponential growth at 0.96, 0.71, 2.17, and 1.40 h^−1^ in M9 medium supplemented with citrate (M9C), M9 medium supplemented with succinate (M9S), and Mueller-Hinton (MH) and Luria-Bertani (LB) media, respectively. The predicted specific growth rates in MH and LB media were higher than those (0.80 and 0.49 h^−1^ for growth in MH and LB, respectively) estimated from early-log (2- to 6-h) growth kinetics (31, 32), because a loose constraint (1 millimole per gram [dry weight] per hour [mmol ⋅ gDW^−1^ ⋅ h^−1^]) was initially set on nutrient uptake reactions to test the growth capability without loss of generality. No growth was predicted on M9 medium supplemented with glucose, which was consistent with the previous phenome results (33). The Biolog assay showed that ATCC 19606 utilized 64 of 190 carbon sources (Fig. 2 and Data Set S1C). Model *i*ATCC19606 predicted bacterial growth on 58 carbon sources and no growth on 122 carbon sources with the overall prediction accuracy achieving 84.3% (Fisher’s exact test, *P = *6.18E−17).

### Prediction of essential genes, reactions, and metabolites for bacterial growth.

**FIG 3:**
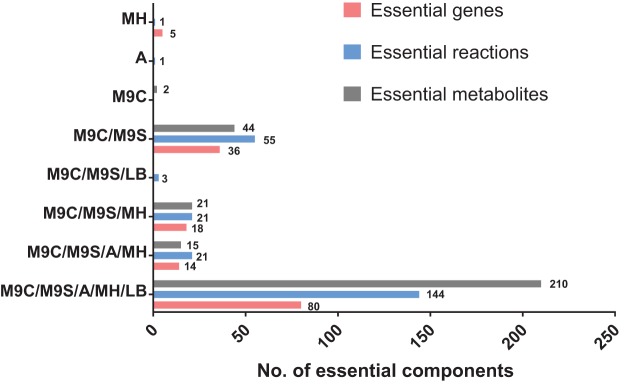
Essential genes, reactions, and metabolites predicted under five nutrient conditions using FBA with the different combinations of media on the *y* axis. The numbers beside bars indicate the number of essential components. M9C, M9 with citrate as the sole carbon source; M9S, M9 with succinate as the sole carbon source; A, arbitrary nutrient; MH, Mueller-Hinton medium; LB, Luria-Bertani medium.

*In silico* single-gene deletion was conducted, and the specific growth rate for each mutant was calculated. With FBA, 148, 148, 80, 117, and 94 genes were predicted to be essential for bacterial growth on M9C, M9S, arbitrary nutrient, and MH and LB media, respectively, whereas using the minimization of metabolic adjustment (MOMA) approach, 157, 157, 93, 126, and 103 essential genes were determined for the above media, respectively (Fig. 3 and Data Set S1D) (34). Across five nutrient conditions, 80 and 88 core essential genes were identified by FBA and MOMA, respectively (Fig. 3 and Data Set S1D), including those from biosynthesis of amino acids, nucleotides, lipids, and cofactors and representing the most indispensable functions for bacterial growth. Comparison with the three-allele transposon mutant library of strain AB5075 grown on LB medium showed a high prediction accuracy of 86.1% by FBA and 85.8% by MOMA (Data Set S1E) (35). Similarly, comparison with the transposon mutant library of ATCC 17978 grown on Vogel-Bonner medium (chemically defined medium with citrate as the sole carbon source, similar to M9C) showed an accuracy of 81.8% by FBA and 82.7% by MOMA (Data Set S1F) (36). Therefore, *i*ATCC19606 is capable of predicting gene essentiality precisely.

Likewise, 244/245, 244/245, 145/145, 149/188, and 168/169 essential reactions were identified by FBA/MOMA when growing on M9C, M9S, arbitrary nutrient, and MH and LB media, respectively (Fig. 3 and Data Set S1G); 292/292, 290/290, 210/212, 246/246, and 225/225 metabolites were considered essential for surviving on the above media by calculating the impaired growth when switching off the consuming fluxes (Fig. 3 and Data Set S1H). Together, 144/144 core essential reactions and 210/212 core essential metabolites were identified by FBA and MOMA, respectively (Fig. 3). High concordance was observed between the core essential components predicted by FBA and MOMA. Specifically, 79/143/210 core essential genes/reactions/metabolites were commonly predicted by the two methods. Gene *nadK* (DJ41_1892, NAD^+^ kinase) and its associated reaction R00104 were identified by FBA as essential, whereas MOMA discovered that *atpIBEFJHAGDC* (DJ41_3203 to DJ41_3211, F_0_F_1_-ATPase), its associated reaction R920, and the cofactor pair ubiquinol/ubiquinone (C00390_c/C00399_c) were essential under all conditions.

### Constraint-based modeling with transcriptomics data predicted metabolic changes in response to colistin treatment.

Gene expression data (Gene Expression Omnibus [GEO] accession no. GSE62794) from the samples of 2-mg/liter colistin treatment and untreated control at 1 h were employed to constrain metabolic fluxes for modeling using the E-Flux method (37). This method simply applies normalized gene expression levels as flux upper bounds to constrain solution space. In responses to colistin treatment, 267 metabolic fluxes in ATCC 19606 were significantly altered (fold change of >2, false discovery rate [FDR] of <0.05; Data Set S1I). Colistin treatment (2 mg/liter) reduced the specific growth rate from 0.82 h^−1^ to 0.57 h^−1^, slightly decreased oxygen uptake (−5.3%) and CO_2_ emission (−3.8%), marginally increased (1.5%) respiration quotient (*q*_CO2_/*q*_O2_), and impaired the uptake of amino acids and dipeptide nutrients (Table 2).

**TABLE 2:** Calculated key fluxes by constraint-based metabolic modeling

Characteristic	Metabolic flux (mmol ⋅ gDW^−1^ ⋅ h^−1^)		FDR
	Control	Colistin (2 mg/liter)	
Biomass formation (h^−1^)	0.82 ± 0.00	0.57 ± 0.00	1.0 × 10^−51^
O_2_ uptake	−45.3 ± 0.02	−42.9 ± 2.44	5.1 × 10^−20^
CO_2_ emission	48.1 ± 0.22	46.3 ± 2.44	2.6 × 10^−12^
Respiratory quotient	1.06 ± 0.00	1.08 ± 0.02	1.8 × 10^−18^
F_0_F_1_-ATPase	73.60 ± 0.21	78.2 ± 3.16	7.3 × 10^−11^
P/O ratio	1.70 ± 0.01	1.87 ± 0.04	2.9 × 10^−13^
Nutrient uptake (mmol of carbon ⋅ gDW^−1^ ⋅ h^−1^)^*a*^	−77.6 ± 0.12	−64.0 ± 0.74	1.3 × 10^−16^

a Calculated by summing up the moles of carbon of each uptaking nutrient.

**FIG 4:**
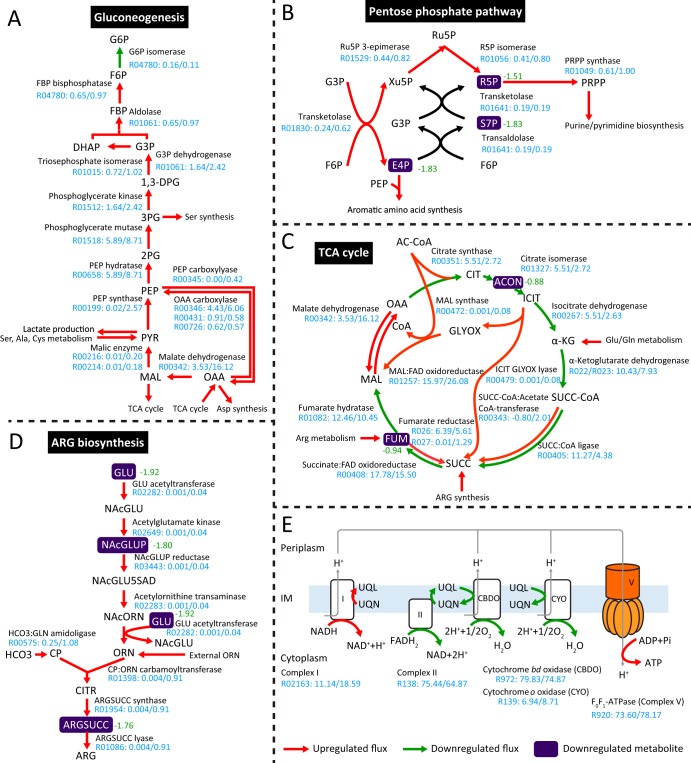
Differentially regulated metabolic fluxes and metabolites in gluconeogenesis (A), pentose phosphate pathway (B), TCA cycle (C), arginine biosynthesis pathways (D), and respiratory chain (E) under 2-mg/liter colistin treatment for 1 h. The specific flux values under control and colistin treatment are denoted in the format flux_control_/flux_colistin_. Significantly altered metabolites from metabolomics data are highlighted in purple with fold change to the side. The metabolite abbreviations are as follows: G6P, glucose 6-phosphate; F6P, fructose 6-phosphate; FBP, fructose 1,6-biphosphate; DHAP, dihydroxyacetone phosphate; G3P, glyceraldehyde 3-phosphate; 1,3-DPG, 1,3-bisphosphoglycerate; 3PG, 3-phosphoglycerate; 2PG, 2-phosphoglycerate; PEP, phosphoenolpyruvate; PYR, pyruvate; MAL, (*S*)-malate; OAA, oxaloacetate; Ru5P, ribulose 5-phosphate; R5P, ribose 5-phosphate; Xu5P, xylulose 5-phosphate; S7P, sedoheptulose 7-phosphate; PRPP, phosphoribosyl pyrophosphate; AcCoA, acetyl-CoA; CIT, citrate; ACON, *cis-*aconitate; ICIT, isocitrate; α-KG, α-ketoglutarate; SUCC-CoA, succinyl-CoA; SUCC, succinate; FUM, fumarate; ARG, l-arginine; GLU, l-glutamate; NAcGLU, *N*-acetyl-glutamate; NAcGLUP, *N*-acetyl-γ-glutamyl-phosphate; NAcGLU5SAD, *N*-acetyl-l-glutamate-5-semialdehyde; NAcORN, *N*-acetyl-ornithine; ORN, l-ornithine; CP, carbamoyl phosphate; HCO_3_, bicarbonate; CITR, l-citrulline; ARGSUCC, argininosuccinate; UQL, ubiquinol-8; UQN, ubiquinone-8; IM, inner membrane.

Within central metabolism, the fluxes through malic enzyme (*maeAB*, DJ41_3218 and DJ41_1760), phosphoenolpyruvate synthase (*ppsA*, DJ41_69), and malate dehydrogenase (*mdh*, DJ41_3006) were upregulated under 2-mg/liter colistin treatment, resulting in a 1.5-fold increase of gluconeogenic flux (Fig. 4A). The end metabolites glyceraldehyde 3-phosphate and fructose 6-phosphate were then utilized by the PPP to generate precursors erythrose 4-phosphate (E4P) and 5-phosphoribosyl 1-pyrophosphate for aromatic amino acid and nucleotide biosynthesis (Fig. 4B). In the TCA cycle, most metabolic fluxes were reduced by 12.7% to 49.3% under colistin treatment, whereas the fluxes over glyoxylate shunt were significantly increased (Fig. 4C).

The biosynthesis of certain amino acids (e.g., l-leucine, l-threonine, l-arginine, and l-lysine) was upregulated after a 1-h treatment with 2 mg/liter colistin. Notably, the l-ornithine uptake (0.003 mmol ⋅ gDW^−1^ ⋅ h^−1^ in the control versus 0.87 mmol ⋅ gDW^−1^ ⋅ h^−1^ after treatment) and *de novo* synthesis were remarkably increased upon colistin treatment. The l-ornithine flux in turn enhanced the production of l-arginine (0.004 mmol ⋅ gDW^−1^ ⋅ h^−1^ in the control versus 0.91 mmol ⋅ gDW^−1^ ⋅ h^−1^ after treatment) (Fig. 4D).

Within oxidative phosphorylation, electron transfer from NADH to ubiquinone (R02163, complex I) increased by 66.8% when cells were treated with 2 mg/liter colistin for 1 h, consistent with the transcriptional upregulation of the *nuoABCDEFGHIJKLMN* operon (DJ41_959-DJ41_947) (37). The overall fluxes via complex II (R138, NADH:flavin adenine dinucleotide [FAD] oxidoreductase), cytochrome *bd* oxidase, and cytochrome *o* oxidase (R972 and R139, respectively) were reduced; however, the ATP generation via complex V (i.e., F_0_F_1_-ATPase) increased by 6% under colistin treatment, owing to the enhanced IM proton potential which possibly resulted from the upregulated cross-IM proton efflux through complex I (Fig. 4E).

### Integrative analysis with metabolomics data using *i*ATCC19606.

**FIG 5:**
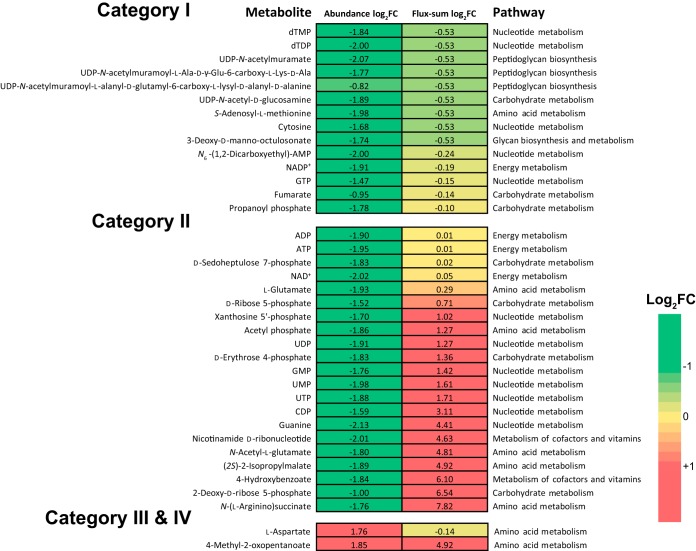
Comparison of flux sums and abundance changes of metabolites caused by 2-mg/liter colistin treatment for 1 h. Category I, metabolites with decreased abundance and flux sums; category II, metabolites with decreased abundance but upregulated flux sums; category III, metabolites with increased abundance but downregulated flux sums; category IV, metabolites with increased abundance and flux sums.

Our previous metabolomics data were integrated with the metabolic flux analysis to further examine the metabolic regulations in response to colistin treatment (38). Among the 103 significantly altered intracellular metabolites, 51 were successfully mapped to *i*ATCC19606, and 37 carried nonzero flux sums (Date Set S1J) and were grouped into four categories: (i) metabolites with decreased abundance and flux sums (14 metabolites), (ii) metabolites with decreased abundance but upregulated flux sums (21 metabolites), (iii) metabolites with increased abundance but downregulated flux sums (l-aspartate only), and (iv) metabolites with increased abundance and flux sums (4-methyl-2-oxopentanoate only, the precursor for l-leucine biosynthesis) (Fig. 5).

The enhanced level of a metabolite could be a result of upregulated influxes and/or downregulated effluxes, whereas downregulated influxes and/or upregulated effluxes would reduce the metabolite pool. Category I represents those depleted metabolites that were putatively due to the repressed biosynthesis activity under colistin treatment. Within the TCA cycle, *cis*-aconitate and fumarate were both downregulated with the decreased flux sums (Fig. 4C and Fig. 5). In the peptidoglycan biosynthesis pathway, the intermediate metabolites UDP-*N*-acetylmuramate, UDP-*N*-acetyl-d-mannosaminouronate, UDP-*N*-acetyl-d-glucosamine, and UDP-*N*-acetylmuramoyl-l-alanyl-d-glutamyl-6-carboxy-l-lysyl-d-alanyl-d-alanine were all downregulated after colistin treatment with the flux sums over these four metabolites decreased by 31.6% (Data Set S1J). Meanwhile, the abundance of LPS inner core biosynthesis precursor 3-deoxy-d-*manno*-octulosonate (KDO) was reduced by 70.5% upon colistin treatment. Consistently, the upstream fluxes via 3-deoxy-d-*manno*-octulosonate 8-phosphate synthase (R03254) and 3-deoxy-d-*manno*-octulosonate 8-phosphatase (R03350) decreased by 31.6% (Data Set S1J). Under colistin treatment, peptidoglycan and lipid A biosynthesis decreased at similar rates because both are essential biomass constituents and were reduced proportionally with bacterial growth declining. The lipid A modifications were neglected here, as metabolic effects could be subtle considering that LPS accounts for only 0.5% (wt/wt) of biomass in the model (Data Set S1A).

Category II contains the metabolites that were potentially drained off due to the increased consuming requirements under colistin treatment. In the PPP, d-ribose 5-phosphate (R5P) and E4P were depleted under 2-mg/liter colistin treatment for 1 h with their flux sums increased by 64.0 and 156.3%, respectively; these results indicated that the metabolite reduction was probably due to the upregulated downstream consuming fluxes toward nucleotide and amino acid biosynthesis (Fig. 4B and Fig. 5). Likewise, the intermediate metabolites *N*-acetyl-l-glutamate, l-argininosuccinate, and (2*S*)-2-isopropylmalate were downregulated, while the associated flux sums were enhanced, probably owing to the increased fluxes toward l-arginine and l-leucine biosynthesis (Fig. 4D and Fig. 5). To test the hypothesis that increasing PPP flux to nucleotide biosynthesis could contribute to intrinsic colistin resistance in A. baumannii, we measured colistin MICs of 7 mutants (Data Set S1K) from the AB5075 transposon library; each of them had a PPP gene disrupted by a transposon insertion. Interestingly, out of the seven mutants, only the *tktB* mutant (AB07666) showed a 4-fold increase of colistin MIC (1 mg/liter versus 0.25 mg/liter for wild-type AB5075). The gene *tktB* (ABUW_2927) encodes the β subunit of transketolase, which catalyzes the transfer of a two-carbon glycolaldehyde unit from a ketose donor (xylulose 5-phosphate) to an aldose acceptor (R5P and E4P). Inactivation of *tktB* would prevent the flux from diverging at R5P and increase the flux toward nucleotide biosynthesis. Hence, colistin in combination with a nucleotide synthesis inhibitor might exert synergistic activity against A. baumannii. Together, the integrative genome-scale metabolic modeling with systems pharmacology data generates key mechanistic insights into colistin activity which may help optimization of polymyxin combination therapy.

## DISCUSSION

The emergence of Gram-negative superbugs that are resistant to the last-resort polymyxins highlights the urgent requirement for novel approaches to understand the complicated mechanisms of antibiotic activity. GSMMs have been increasingly employed to decipher antibiotic killing and resistance (39–41), optimize combination therapy (42), and design novel treatments (43). Here we report the development, validation, and application of a GSMM designated *i*ATCC19606 for the type strain A. baumannii ATCC 19606. This strain was originally isolated from a patient urine sample and has been widely used in microbiological and pharmacological studies (44–46). Modeling with transcriptomics and metabolomics data using *i*ATCC19606 revealed a broad range of interesting metabolic changes in response to 2-mg/liter colistin treatment at 1 h, including (i) upregulated gluconeogenesis, PPP, and amino acid biosynthesis; (2) downregulated TCA cycle and peptidoglycan and LPS biosynthesis; and (3) altered respiratory activities and energy generation.

Until now, there have been only three GSMMs available for A. baumannii, i.e., AbyMBEL891 (23), iLP844 (25), and iCN718 (24). Models AbyMBMEL891 and iCN718 were constructed specifically for strain AYE. Reciprocal BLASTp showed significant genomic discrepancies between AYE and ATCC 19606; ATCC 19606 contains 606 of 3,669 (16.5%) unique genes, whereas AYE has 593 of 3,703 (16.0%) unique genes. Importantly, the mechanisms of acquiring polymyxin resistance were well studied using ATCC 19606 (11, 47); therefore, an ATCC 19606-specific model was developed in this study. The recently published model iLP844 and our *i*ATCC19606 share 679 reactions and contain 949 and 591 unique reactions, respectively (see Data Set S1L in the supplemental material). The differences between the two models were mainly due to using different compartment settings, transport, and exchange reactions. In addition, for iLP844, only 67 nutrients (actually 66 according to their supplemental material) were tested for the validation (25). In contrast, our model *i*ATCC19606 predicted bacterial growth on 190 carbon substrates and reached equivalent accuracy of 84.3% (Fig. 2 and Data Set S1C). For the 66 metabolites that iLP844 used (25), the prediction with our *i*ATCC19606 achieved a higher accuracy of 86.3% (Data Set S1C). *i*ATCC19606 and iLP844 share 45 essential genes, representing 48.4% (*i*ATCC19606) and 67.2% (iLP844) of the total essential gene lists, respectively (Data Set S1D). Previous essentiality analysis with iLP844 showed an overall prediction accuracy of 80.9%, with only 42 essential genes being correctly predicted (25). Using the same mutant library of strain ATCC 17978 as the reference, our *i*ATCC19606 showed a prediction accuracy increased up to 82.7% (Data Set S1F), possibly owing to different gene associations between the two GSMMs. For instance, thymidylate synthase (*thyA,* DJ41_1187), catalyzing the conversion from dUMP to deoxythymidine monophosphate, was demonstrated to be essential for A. baumannii growth (35, 36). This gene was absent in iLP844 but was included in *i*ATCC19606 and correctly predicted as essential. Gene *cyoD* (DJ41_64, encoding cytochrome *o* ubiquinol oxidase subunit IV) was predicted as nonessential in iLP844, whereas our prediction classified *cyoD* (ABUW_1550 in the AB5075 genome) as essential, which agrees with the absence of a corresponding mutant in A. baumannii transposon libraries (35, 36).

Model iLP844 was integrated with transcriptomics data of 2-mg/liter colistin treatment and untreated control samples at 1 h to simulate the metabolic responses to colistin, by simply converting continuous gene expression to binary states using the MADE algorithm, with 0 indicating downregulation and 1 indicating upregulation (25). Hence, the flux-carrying capacity for a reaction could be either zero or the maximum value. This discretization could cause coarse-grained representation of gene expression and thus affect the prediction accuracy. For instance, the expression of isocitrate lyase-encoding gene DJ41_2528 was at a low level in the untreated control (135.3 ± 13.6 in reads per kilobase of transcript per million mapped reads [RPKM]; GEO accession no. GSE62794) and upregulated by 2.2-fold after 2-mg/liter colistin treatment for 1 h (37). Implementation of the MADE algorithm completely shut down the associated reaction rxn00336_c0 (25), whereas nonzero fluxes (R00479, 0.002 and 0.08 mmol · gDW^−1^ · h^−1^ for control and colistin treatment, respectively [Data Set S1I]) were obtained using E-Flux. Furthermore, most flux upper bounds in iLP844 were set to the maximum capacity by MADE, resulting in an overestimated growth rate (i.e., 36.4 h^−1^ and 42.7 h^−1^ for the control and colistin treatment at 1 h, respectively), whereas previous experimental results showed that A. baumannii usually grew at approximately 0.80 h^−1^ in MH medium (31). In addition, the modeling of iLP844 employed only transcriptomics data as flux constraints. As biological systems regulate metabolic responses at multiple levels (48, 49), lack of experimental validation from the metabolomic level may compromise the prediction reliability. Having incorporated transcriptomic constraints using the E-Flux method (17), our modeling maintained the continuous nature of gene expression changes (Data Set S1I). Furthermore, metabolomics data from the same condition (38) were also employed for integrative analyses in our study (Data Set S1J). Therefore, our integrative modeling with multiomics data provides a comprehensive understanding from both transcriptional and metabolic perspectives.

Bacterial metabolism plays a vital role in mediating the cellular responses to treatments with antibiotics (50), such as the polymyxins (38). Individual omics approaches (e.g., transcriptomics and metabolomics) have revealed that many cellular processes participated in the response to polymyxins, including cellular respiration, carbon catabolism, iron homeostasis, amino acid and nucleotide biosynthesis, and redox balance (38, 47, 51). However, major gaps exist between transcriptomics and metabolomics, such as (i) changes in gene expression do not necessarily induce alterations of metabolic activity and (ii) changes in metabolite intensity do not necessarily indicate the results of transcriptional regulation. Hence, we developed *i*ATCC19606 to bridge the gaps between transcriptomics and metabolomics. By imposing the transcriptomic constraints, *i*ATCC19606 allowed simulating reaction flux and metabolic network utilization for untreated and colistin-treated A. baumannii, providing a global view of metabolic responses to antibiotic killing (Fig. 4 and Data Set S1I). Integrative analysis of the metabolic fluxes and metabolomics further identified meaningful regulations between fluxes and metabolites in response to colistin treatment (Fig. 5 and Data Set S1J). For example, our simulation results showed a decreased flux via LPS biosynthesis, consistent with the 1.9-fold-downregulated expression of *lpxA* (DJ41_250) and 3.3-fold-decreased level of LPS inner core precursor KDO (38). Gram-negative bacterial OM fortifies the cell against environmental stresses, and A. baumannii can develop colistin resistance via loss of LPS (52, 53). For peptidoglycan biosynthesis, the metabolic fluxes and metabolomics data collectively showed significant downregulation, indicating that less peptidoglycan was synthesized under colistin treatment (Data Set S1I).

Within amino acid biosynthesis, the upregulation of amino acid *N*-acetyltransferase (DJ41_3725, *argA*, 7.6-fold) and acetylglutamate kinase (DJ41_2686, *argB*, 2.0-fold) resulted in upregulated arginine biosynthesis flux and a decreased intermediate metabolite pool of *N*-acetyl-l-glutamate (3.5-fold) and l-argininosuccinate (3.4-fold, Fig. 4D). The acetylglutamate synthesis function of ArgA can be complemented by ornithine *N*-acetyltransferase (*argJ*, DJ41_1001), which recycles the acetyl group from acetylornithine to glutamate (54). It has been found that the ArgJ-mediated arginine biosynthesis pathway played a key role in the persistence of Staphylococcus aureus after gentamicin treatment (55). The upregulated expression of *argAB* was also reported in LPS-loss mutants of A. baumannii ATCC 19606, indicating that enhanced arginine biosynthesis is a conserved response to colistin in A. baumannii (14). Together, A. baumannii may upregulate arginine biosynthesis to increase cell survival during colistin action via (i) production of ammonia which mitigates against toxic hydroxyl radicals and (ii) generation of polyamines to attenuate the colistin-LPS electrostatic interaction in the OM (56).

In the TCA cycle (Fig. 4C), most of the genes were upregulated after 2-mg/liter colistin treatment at 1 h, including citrate synthase (DJ41_3568, 1.5-fold), aconitate hydratase (DJ41_1103, 2.7-fold), 2-oxoglutarate dehydrogenase (DJ41_3573 and DJ41_3574, 1.9 and 2.5-fold, respectively), succinyl-CoA ligase (DJ41_3576 and DJ41_3577, 2.1-fold for both), fumarate hydratase (DJ41_227, 2.6-fold), and malate dehydrogenase (DJ41_3006, 1.8-fold). However, the fluxes and metabolites throughout the TCA cycle decreased. In contrast, upregulated malate synthase (DJ41_669, 1.6-fold) and isocitrate lyase (DJ41_2528, 2.2-fold) resulted in increased fluxes over glyoxylate shunt, bypassed the lower TCA cycle, and increased flux via malate dehydrogenase (DJ41_3006, 1.8-fold). A previous study discovered that stalled TCA cycle and enhanced glyoxylate shunt conferred Pseudomonasaeruginosa cell tolerance to aminoglycoside antibiotics (57). Our observation indicates that A. baumannii may also upregulate glyoxylate shunt in response to colistin treatment.

The metabolic changes characterized above are likely to occur in colistin-susceptible strains under colistin treatment. For instance, 2-mg/liter polymyxin B treatment for 2 h induced significant depletion of intermediate metabolites in the PPP and TCA and nucleotide biosynthesis pathway in ATCC 17978 (31), similarly to that in ATCC 19606 (38). These metabolic changes could be essential for bacterial responses to polymyxin treatment in susceptible strains. Interestingly, depletion of the intermediate metabolites in the PPP and TCA also occurred in LPS-deficient, polymyxin-resistant 19606R, compared to its paired polymyxin-susceptible ATCC 19606 (15). This may indicate that the adaptation to metabolic changes in these pathways might contribute to polymyxin resistance in 19606R.

### Conclusions.

We have constructed and validated a GSMM, *i*ATCC19606, for type strain A. baumannii ATCC 19606. Importantly, integrative modeling with correlative transcriptomic and metabolomic data provided, for the first time, key mechanistic insights into metabolic responses to colistin treatment in A. baumannii. Our metabolic model can be used as a powerful tool for systematically assessing key genes and metabolic pathways that contribute to bacterial responses to antibiotic treatment and elucidation of the molecular mechanisms. Combined with antibiotic pharmacokinetics/pharmacodynamics, *i*ATCC19606 will be able to predict the time course of bacterial responses to antibiotic treatment at the network level in A. baumannii and provides an *in silico* platform for developing precision antimicrobial therapy.

## MATERIALS AND METHODS

### Strains and media.

A. baumannii ATCC 19606 was obtained from the American Type Culture Collection and cultured on nutrient agar (NA) and in cation-adjusted Mueller-Hinton broth (CaMHB) and Luria-Bertani (LB) medium. Tryptic soy broth containing 20% (vol/vol) glycerol was used for stock at −80°C.

### Construction of a genome-scale metabolic model.

The genome assemblies and annotations of A. baumannii AYE (GenBank accession numbers CU459137 to CU459141) and ATCC 19606 (GenBank accession numbers ACQB01000000 and JMRY00000000) were obtained from the GenBank database. Reciprocal BLASTp was implemented to determine the orthologs between AYE and ATCC 19606, with sequence identity of >70%, E value of <1E−5, and coverage of >70% (28). The existing model AbyMBEL891 for AYE (23) was employed as a starting template to expedite model construction. Specifically, for each reaction in AbyMBEL891, the associated genes were replaced with the corresponding orthologs in ATCC 19606. The reactions without any orthologs in ATCC 19606 were considered AYE specific and removed from the reaction list. Isolated metabolites were removed as well, followed by supplementation of missing metabolites, reactions, and genes according to the genome annotation of ATCC 19606 and biochemical databases KEGG (58) and MetaCyc (30). Extensive manual curation was conducted, including (i) adding transport reactions and extracellular metabolites, (ii) filling pathway gaps, and (iii) checking the mass and charge balance of each reaction. The resulting model was compiled in Systems Biology Markup Language (59), and VANTED (60) was employed for metabolic network visualization and analysis. The Memote test suite (61) was used to confirm that the constructed model met the basic standards for consistency, formatting, and reusability. The biomass formation equation from AbyMBEL891 was used in the present study.

### Biolog assay and prediction of nutrient utilizations.

ATCC 19606 was streaked out from glycerol stock on NA and subcultured at 37°C for 20 h. Bacterial cells were swapped into a sterile capped tube containing 16 ml IF-0 solution (Cell Biosciences, West Heidelberg, Australia) until the turbidity reached 42% transmittance in a turbidimeter (Pacificlab, Blackburn, Australia). The cell suspension was then diluted 5 times with IF-0 solution and dye (Cell Biosciences) to final 85% transmittance. Biolog phenotype microarrays (PMs) 1 and 2 (Cell Biosciences) were employed to test the utilization of 190 carbon sources, with two independent biological replicates. Bacterial growth was detected after 18 and 24 h of incubation at 37°C by measuring the optical density at 595 nm using an Infinite M200 microplate reader (Tecan, Mannedorf, Switzerland). Readings of ≥1.3-fold of blank medium controls indicated the utilization of nutrients.

The constructed GSMM *i*ATCC19606 was then employed to predict the bacterial growth on a chemically defined medium with 190 individual carbon sources using the FBA method (62). Biomass formation was maximized with the maximum specific carbon nutrient uptake rate set at 1 mmol ⋅ gDW^−1^ ⋅ h^−1^ under aerobic conditions.

$$\max\;v_{\text{biomass}}$$

s.t. S⋅v=0

$$a_{j}\leq v_{j}\leq b_{j},j=1,2,\cdots ,n$$

where *S* represents the stoichiometric matrix with *m* metabolites and *n* reactions. Each flux *v_j_* is constrained by the lower bound *a_j_* and upper bound *b_j_*. The calculated growth phenotypes were compared with Biolog results to assess prediction accuracy using a commonly accepted method (63). Specifically, a correct prediction of a utilizable or nonutilizable carbon source for growth in LB was considered a true positive (TP) or true negative (TN), respectively, whereas an incorrect prediction of a utilizable or nonutilizable carbon source was considered a false negative (FN) or false positive (FP). The overall accuracy of prediction was then evaluated by accuracy = (TP + TN)/(TP + TN + FP + FN). The significance was evaluated by Fisher’s exact test.

### Gene essentiality analysis.

*In silico* single-gene deletion was conducted using both FBA and MOMA (34) algorithms. Minimization of metabolic adjustment (MOMA) was developed to predict the metabolic flux redistribution in a gene knockout mutant. MOMA hypothesizes that the metabolism of the mutant tends to approximate the wild type (34). Therefore, MOMA was employed to calculate gene essentiality as a complementation of the FBA method. Nutrient uptake constraints were set to M9, arbitrary nutrient, MH, and LB media (64) (see Data Set S1M in the supplemental material). Likewise, essential reactions and metabolites were predicted by calculating the growth rate when switching off either the corresponding reaction or consuming fluxes, respectively (65). The recently generated three-allele transposon mutant library for A. baumannii AB5075 (35) and transposon library for ATCC 17978 (36) were employed as references to assess the prediction accuracy. Specifically, a correct prediction of an essential or nonessential gene for bacterial growth in LB medium was considered a true positive (TP) or true negative (TN), respectively, whereas an incorrect prediction of an essential or nonessential gene was considered a false negative (FN) or false positive (FP). The overall accuracy of prediction was then evaluated by accuracy = (TP + TN)/(TP + TN + FP + FN). The significance was evaluated by Fisher’s exact test. The two mutant libraries were employed for validation, because to the best of our knowledge, they are the only A. baumannii libraries providing gene essentiality information. BLASTp was performed to identify the orthologs between AB5075, ATCC 17978, and ATCC 19606, with the criteria of sequence identity of >70%, Expect value (E-Val) of <1E−5, and coverage of >70% (28). Totally, 453 and 398 essential genes were identified for the two strains, respectively (35, 36); between the two, 168 common essential orthologs were discovered.

### Prediction of metabolic responses to colistin treatment by constraining metabolic fluxes with transcriptomics data.

The RNA-Seq data (GEO accession number GSE62794) for ATCC 19606 growth in CaMHB with the absence (control) and presence of 2 mg/liter colistin (treatment) at 1 h were employed as flux constraints using the E-Flux algorithm (17); these transcriptomics data were used only for modeling metabolic responses to colistin treatment. For each gene in the model, the RPKM value was calculated using the edgeR package (66) and normalized to constrain the flux upper limits using the E-Flux algorithm (17). The maximum uptake rates of amino acids, vitamins, and dipeptides in *i*ATCC19606 were set to 1 mmol ⋅ gDW^−1^ ⋅ h^−1^ (67), given that in CaMHB these nutrients serve as the major carbon sources for bacterial growth. For either the control or colistin treatment condition, the metabolic solution space was sampled with 10,000 random points using the ll-ACHRB (loopless Artificially Centered Hit-and-Run on a Box) algorithm, by allowing the growth rate varying within 99 to 100% of its maximum value (68). Statistical significance of differential flux distributions was computed using the Z-score method with FDR of <0.01 and fold change of >2 (69). FDR represents the false discovery rate calculated from the *P* value using the Benjamini-Hochberg method (70). This method was used to control the false discovery rate when conducting multiple comparisons. The metabolic fluxes with dramatic flux changes were then referred to the metabolic pathways from KEGG and MetaCyc for further analyses.

### Integrative analysis with correlative metabolomics data.

Our liquid chromatography-mass spectrometry (LC-MS)-based untargeted metabolomics data of ATCC 19606 (38) were employed for integrative analysis of metabolic responses. ATCC 19606 (10^8^-CFU/ml inoculum) was aerobically grown in CaMHB at 37°C, in the absence (control) and presence of 2 mg/liter colistin (treatment). Intracellular metabolomics data from the control and treatment at 1 h (*n *=* *4) were employed in this study. A total of 1,535 and 1,526 unique metabolites were putatively identified for the control and treatment conditions at 1 h, respectively. One-way ANOVA (analysis of variance) was used to determine differentially abundant metabolites, with criteria of fold change of ≥1.5, *P* of ≤0.05, and FDR of ≤0.1. For each intracellular metabolite in *i*ATCC19606, the relative abundance change after 2-mg/liter colistin treatment at 1 h was correlated with the calculated flux sums (i.e., turnover rates) (71), to analyze how metabolic flux changes resulted in metabolite pool alterations under colistin treatment. For each obtained random solution, flux sums (φ) were calculated by summing up all producing fluxes (influxes) or consuming fluxes (effluxes) at metabolite nodes (71).

$$\varphi_{i}=\overset{n}{\underset{j}{\sum }}\left|S_{ij}v_{j}\right|,i=1,2,\cdots ,m$$

where φ*_i_* represents the *i*th metabolite in *i*ATCC19606. Differentially altered flux sums were identified using the above Z-score methods with FDR of <0.01 (69). Metabolites with significantly changed abundance and altered flux sums were selected for downstream analysis. Specifically, the reduced metabolite pool could be a consequence of decreased production or increased consumption of that metabolite, whereas the increased metabolite pool could be caused by increased production or decreased consumption of that metabolite.
